# Algorithmic Modeling Quantifies the Complementary Contribution of Metabolic Inhibitions to Gemcitabine Efficacy

**DOI:** 10.1371/journal.pone.0050176

**Published:** 2012-12-11

**Authors:** Ozan Kahramanoğullari, Gianluca Fantaccini, Paola Lecca, Daniele Morpurgo, Corrado Priami

**Affiliations:** 1 The Microsoft Research–University of Trento Centre for Computational and Systems Biology, Rovereto (Trento), Italy; 2 Department of Mathematics, University of Trento, Povo (Trento), Italy; Memorial Sloan Kettering Cancer Center, United States of America

## Abstract

Gemcitabine (2,2-difluorodeoxycytidine, dFdC) is a prodrug widely used for treating various carcinomas. Gemcitabine exerts its clinical effect by depleting the deoxyribonucleotide pools, and incorporating its triphosphate metabolite (dFdC-TP) into DNA, thereby inhibiting DNA synthesis. This process blocks the cell cycle in the early S phase, eventually resulting in apoptosis. The incorporation of gemcitabine into DNA takes place in competition with the natural nucleoside dCTP. The mechanisms of indirect competition between these cascades for common resources are given with the race for DNA incorporation; in clinical studies dedicated to singling out mechanisms of resistance, ribonucleotide reductase (RR) and deoxycytidine kinase (dCK) and human equilibrative nucleoside transporter1 (hENT1) have been associated to efficacy of gemcitabine with respect to their roles in the synthesis cascades of dFdC-TP and dCTP. However, the direct competition, which manifests itself in terms of inhibitions between these cascades, remains to be quantified. We propose an algorithmic model of gemcitabine mechanism of action, verified with respect to independent experimental data. We performed in silico experiments in different virtual conditions, otherwise difficult in vivo, to evaluate the contribution of the inhibitory mechanisms to gemcitabine efficacy. In agreement with the experimental data, our model indicates that the inhibitions due to the association of dCTP with dCK and the association of gemcitabine diphosphate metabolite (dFdC-DP) with RR play a key role in adjusting the efficacy. While the former tunes the catalysis of the rate-limiting first phosphorylation of dFdC, the latter is responsible for depletion of dCTP pools, thereby contributing to gemcitabine efficacy with a dependency on nucleoside transport efficiency. Our simulations predict the existence of a continuum of non-efficacy to high-efficacy regimes, where the levels of dFdC-TP and dCTP are coupled in a complementary manner, which can explain the resistance to this drug in some patients.

## Introduction

Gemcitabine (2,2-difluorodeoxycytidine, dFdC) is a prodrug, which is commonly used in the treatment of patients with non-small-cell lung cancer, pancreatic cancer, bladder cancer, and breast cancer. It is currently the leading therapeutic for pancreatic ductal adenocarcinoma treatment [1]–[3]. Gemcitabine is also used in the treatment of relapsed or refractory low-grade non-Hodgkin's lymphoma and, in combination with other drugs, in lymphatic and myeloid malignancies [4]. Gemcitabine occupies a prominent place as a chemotherapeutic agent. However, for a majority of patients the response rate following its administration with respect to stability of disease is subject to resistance [1], [5]. A better understanding of the mechanisms of resistance to gemcitabine is thus important in cancer treatment, also due to the lack of clinically effective markers for predicting which patient will benefit from treatment.

**Figure 1:**
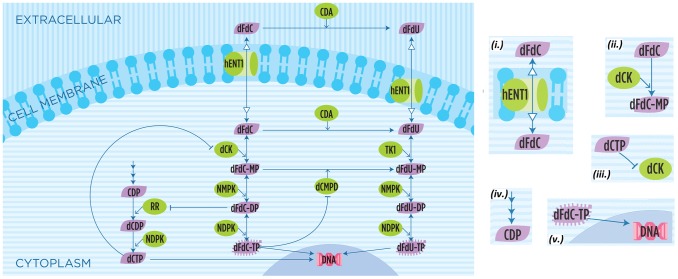
Gemcitabine biochemical machinery. The biotransformation and pharmacologic action of dFdC and its metabolites. The different arrows, with respect to the legend on the right, are (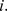) transport into cell (by a nucleoside transporter hENT1); (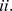) enzymatic reaction (where dCK is the enzyme); (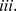) inhibition (of dCK by binding with dCTP); (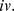) synthesis (of CDP); (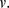) DNA incorporation (of dFdC-TP). See the text for or a more detailed description.

Gemcitabine is a nucleoside analog in which the hydrogen atoms on the 2′-carbon of deoxycytidine are replaced by fluorine atoms. Gemcitabine is metabolized to exert its clinical action [6], whereby it is transformed into its triphosphate metabolite dFdC-TP, and into its deaminated uracil triphoshate metabolite dFdU-TP. Gemcitabine efficacy is mainly attributed to dFdC-TP, as the uridine metabolite is largely excreted into the urine [2], while recent evidence suggests partial contribution of dFdU-TP to cytotoxicity [7]. The cytotoxic effect of gemcitabine on tumor cells is realized by the inhibition of the DNA synthesis. As with fluorouracil and other analogues of pyrimidines [8], the triphosphate analogue dFdC-TP replaces one of the building blocks of nucleic acids, in this case cytidine, during DNA replication. As only one additional nucleoside can be attached to the “faulty” nucleoside, this prevents cells from processing DNA, thereby blocking the cell cycle in the early S phase and causing apoptosis [9]. The incorporation of dFdC-TP takes place in competition with the natural nucleoside dCTP, which also incorporates into DNA. As a consequence, effective functioning of the cytotoxic mechanism is enhanced by the depletion of the dCTP pools, which is also attributed to gemcitabine efficacy (Figure 1).

In-vitro studies on pharmacokinetic properties of gemcitabine indicate that resistance to this drug may result from multiple factors [5]. Efficient uptake is a requirement for anticancer efficacy as it has been addressed by monitoring sensitivity in a variety of established lymphoid cell lines with defined nucleoside transporter activities and in tissue samples from pancreatic ductal adenocarcinoma patients treated with gemcitabine [6], [10], [11]. In this respect, it has been suggested that diminished expression or activity of nucleoside transporters, e.g., human equilibrative nucleoside transporter 1 (hENT1) [12] and concentrative nucleoside transporter 1 (hCNT1) [13], could lead to reduced activity of anticancer nucleosides both in vitro and in vivo. Although transport across the plasma membrane is essential, a rate-limiting step in gemcitabine activation is its phosphorylation by deoxycytidine kinase (dCK) [14], [15]. The correlation between dCK levels and gemcitabine sensitivity can thus be considered as a prognostic parameter in gemcitabine therapy [11], [12], [16]. However, the activity of dCK depends on other factors such as the inhibition of this enzyme by dCTP. In return, phosphorylated gemcitabine depletes the dCTP pool by inhibiting the enzyme ribonucleotide reductase (RR) [17]–[19].

The indirect competition, that is, the competition for common resources, between dFdC-TP and dCTP for integration into DNA is an efficacy parameter. In this respect, the expression of the metabolite genes that are involved in the gemcitabine metabolism plays an important role in determining efficacy [3], [12]. However, the inhibitions between synthesis cascades of the metabolites dFdC-TP and dCTP also have a direct influence on each other's levels by exerting a mechanism of direct competition, where RR and dCK play a dual role, inhibiting or increasing the accumulation of the two metabolites. The phenomena that result in impaired drug responsiveness are thus interconnected with factors such as deficiency in dCK, increased dCTP pools, and decreased influx into cell [20], [21], quantification of which are blurred in the literature. In particular, despite a plethora of articles on gemcitabine pharmacodynamics and pharmacokinetics, little is known regarding the interplay within the metabolic network that exerts an inhibitory effect on gemcitabine and the inhibitory effects exerted by gemcitabine on the network [4], [22].

We investigate the contribution of the inhibitory mechanisms to the gemcitabine machinery. For this purpose, we present an algorithmic model that describes the intracellular metabolic network that involves gemcitabine and its main metabolites. We use continuous time discrete stochastic simulations, which provide an account of the random fluctuations due to the variability of the conditions in the biochemical environment [23]–[25]. This allows us to give an algorithmic interpretation [26] of the experimental data mechanistically with respect to the time series concentrations [7] and efficacy measurements [27].

Following the intuition of Nobel laureate Nurse [28], we think that a language-based computational approach is adequate for modeling complex biological systems, because it brings about an ease in extending and managing the models. The algorithmic representations of biological systems [26] are then amenable to computer execution, and the state changes of computer programs can be directly mapped to dynamic state changes of biological networks [24], [25], [29]. Language technology and compiler theory provide the means to build high-level abstractions, comprehensible to non expert modelers, to be mapped onto executable languages [30]–[32]. This allows computational and wet-lab experts to easily dialogue over a model and map it to a desired setting for simulation and analysis.

In agreement with the experimental data, our model indicates that the inhibitions due to the association of dCTP with dCK and the association of dFdC-DP with RR play a key role in adjusting the efficacy. While the former tunes the catalysis of the rate-limiting first phosphorylation of dFdC, the latter is responsible for depletion of dCTP pools, thereby providing higher efficacy. Our simulations predict the existence of a continuum of non-efficacy to high-efficacy regimes where the levels of the metabolites dFdC-TP and dCK are coupled in a complementary manner, which can explain the resistance to this drug in some patients. Our model indicates that nucleoside transport efficiency is essential for efficacy, whereas the complementarity of the dCK and dFdC-TP metabolite levels is a function of the association of dFdC-DP with RR. Due to the modeled mechanisms that are conserved among various cancers and the explicit stochasticity, which provides a representation of the intrinsic noise due to genetic variations in different patients, our model can also serve as a general tool to study gemcitabine resistance in specific clinical applications, where the model can be further enriched with relevant genetic parameters.

## Results

We developed an algorithmic model [26] describing the intracellular metabolic network that involves gemcitabine and its main metabolites, depicted in Figure 1. The model describes the following machinery.

Gemcitabine is transported into cells by equilibrative and concentrative nucleoside transporters [6], [13], e.g., human equilibrative nucleoside transporter 1 (hENT1) and human concentrative nucleoside transporter 1 (hCNT1). It is then phosphorylated by deoxycytidine kinase (dCK) to its monophosphate dFdC-MP. It is subsequently phosphorylated to its active metabolites dFdC-DP and dFdC-TP with the intervention of nucleoside monophosphate kinase (NMPK) and nucleoside diphosphate kinase (NDPK), respectively. Gemcitabine exerts its effect by two main mechanisms: while the diphosphate metabolite dFdC-DP plays an inhibitory role for the synthesis of natural nucleoside triphosphate dCTP, the triphosphate metabolite dFdC-TP competes with the dCTP for incorporation into nascent DNA chain, thereby inhibiting DNA synthesis and blocking cells in the early DNA synthesis phase. That is, in a competing pathway, while dCTP inhibits dCK [33]–[35], dFdC-DP inhibits ribonucleotide reductase RR in an irreversible manner [4], [36], [37], whereby it eventually depletes the dCTP pool, decreasing the dCK inhibition and facilitating the DNA incorporation of dFdC-TP. A competing and inactivating pathway is triggered with the rapid deamination of dFdC by cytidine deaminase (CDA) to 2,2-difluorodeoxyuridine dFdU. Alternatively, dFdC-MP is converted to dFdU-MP by deoxycytidylate deaminase (dCMPD) whereas dCMPD is inhibited by dFdC-TP [38]. dFdU is transported into cells by nucleoside transporters and phosphorylated to its monophosphate dFdU-MP, diphosphate dFdU-DP and its triphosphate dFdU-TP, whose activity has been recently associated with the cytotoxic effect of the drug [7].

In the following, we abbreviate the inhibitions of the metabolic network as follows: (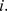) dCK inhibition refers to the inhibition due to the association of dCK and dCTP; (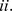) RR inhibition refers to the inhibition due to the association of RR and dFdC-DP; (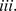) dCMPD inhibition refers to the inhibition due to the association of dCMPD and dFdC-TP.

### Gemcitabine efficacy is correlated with the dCK inhibition propensity

The biochemical mechanism of gemcitabine mimics the cascade that results in the incorporation of dCTP to DNA, while competing with it for incorporation into DNA. The two cascades interact with each other by means of the dCK inhibition and RR inhibition. It is known that dCK is the rate-limiting enzyme in the gemcitabine activation cascade due to its role in the first phosphorylation [14], [15]. We ran simulations and measured the effect of the inhibitory mechanism to gemcitabine efficacy. Following [7], we assumed that efficacy is proportional to the area under the curve (AUC) of the dFdC-TP in the simulations for the 24 hours after the administration of the drug. Similarly, we assumed that dCTP levels relative to dFdC-TP during the simulation is an efficacy determinant, thus AUC of dCTP provides a measure of efficacy as well. These assumptions provide observations that are consistent with the experiments reported in [27] (see Materials and Methods, Figures 6, and Figure 7).

In order to estimate the effect of all three inhibitions to dFdC-TP accumulation, we performed simulations by varying their rates, in isolation and in combination with others. We first considered the contribution of the individual inhibitions in isolation within a spectrum of 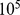 to 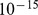 with the unit of measure 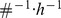 for the association rates and 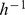 for the dissociation rates. Because the rates of these inhibitions can depend on many factors, these experiments model variations in the propensities of these inhibitions due to metabolic conditions. We scaled down the initial number of molecules by three orders of magnitude, and measured the AUC of the metabolites in order to factor for the intrinsic noise [23].

**Figure 2:**
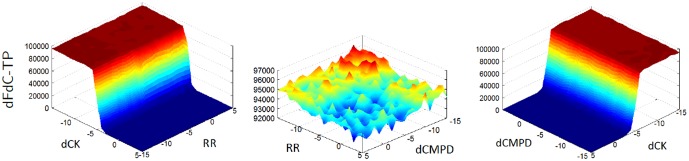
Sensitivity to paired inhibitions. The plots displaying the area under the curve (AUC) for dFdC-TP that result from the simulations where the association rates of the inhibitions are varied from 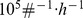 to 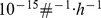, given in logarithmic scale. The dissociation rates for the inhibitions are set to 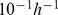. At the simulations for the plot on the left, dCMPD inhibition rate is set to zero, and the dCK and RR are varied. At the middle plot, dCK inhibition rate is set to zero, and at the plot on the right RR inhibition rate is set to zero.

Our simulations, depicted in Figure S2 in the supplementary material, show that the propensity of the dCK inhibition plays an important role in adjusting the dFdC-TP levels, hence the gemcitabine efficacy. This observation emphasizes the role played by dCK inhibition in determining the dFdC-TP levels, and the contribution of dCTP accumulation as a factor with direct influence on the first phosphorylation of dFdC. This also indicates that other factors that are not included in our model can however determine gemcitabine efficacy by influencing the dCK-dCTP association affinity. The experiments, where we fixed the unbinding rates to 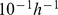, and considered the contributions of pairs of inhibitions, indicated that the RR and dCMPD inhibitions have a minor effect on dFdC-TP accumulation, in particular with respect to dCK inhibition. However, their effect is cumulative as demonstrated in Figure 2.

### dFdC-TP and dCTP levels complement each other with RR inhibition

Gemcitabine exerts its clinical effect by incorporating its triphosphate metabolite dFdC-TP into DNA in competition with the natural nucleoside triphosphate dCTP. We distinguish between direct and indirect competition in determining efficacy associated with the cytotoxic effect of gemcitabine: the indirect competition between dFdC-TP and CTP is given by the race for incorporation into DNA as they compete for common resources. We define the direct competition mechanism with the dCTP depletion due to inhibitory interactions between the cascades that result in dFdC-TP and dCTP. Since RR plays an important role in the synthesis of dCTP, its inhibition by dFdC-DP is a direct competition mechanism that controls the dCTP accumulation.

**Figure 3:**
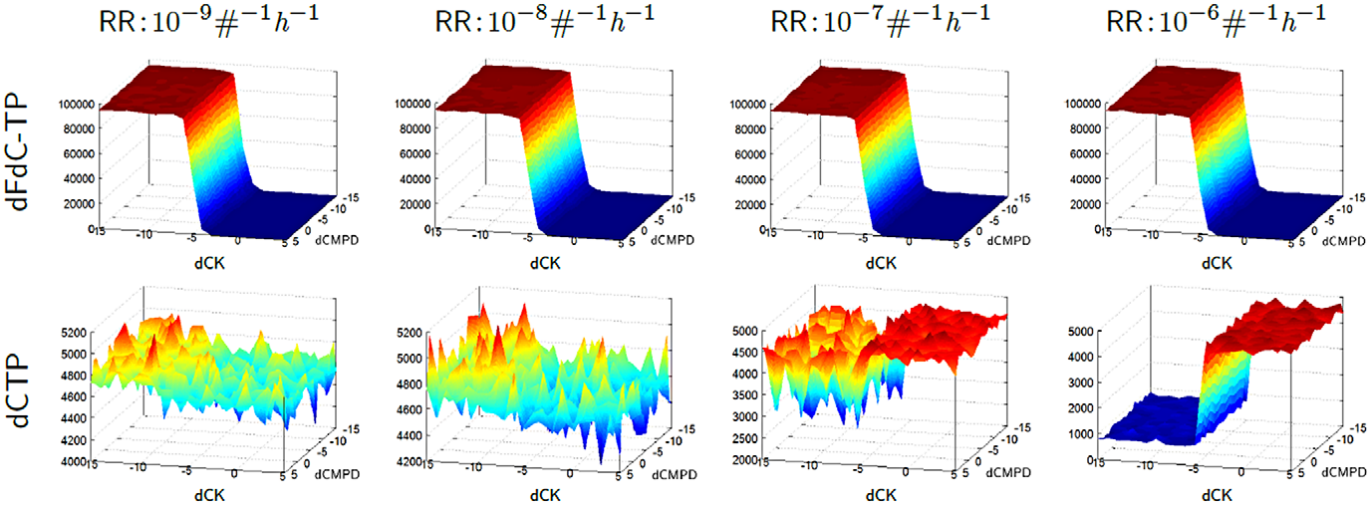
Sensitivity to dCK, dCMPD and RR inhibitions. The plots displaying the area under the curve (AUC) for dFdC-TP (top row) and dCTP (bottom row) that result from the simulations with respect to different rate values. At every column, there are plots for different RR inhibition association rates, varied from 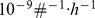 to 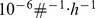 with an order of magnitude at each step. In each plot, the association rates of the dCK and dCMPD inhibitions are varied from 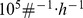 to 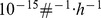, given in logarithmic scale. The dissociation rates are set to 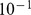. A scaling factor of 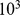 is used for intrinsic noise, which is observed at the dCTP plots where RR inhibition rate values are less than 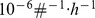.

In order to estimate the effect of all three inhibitions to dCTP accumulation relative to dFdC-TP, we compared the AUC of these metabolites during simulations, where the inhibition rates are varied as depicted in Figure 3. Our simulations indicate that dCTP accumulation is insensitive to dCMPD inhibition. While a decrease in RR inhibition propensity has a significant positive effect to dCTP accumulation, a propensity for the RR inhibition, which is greater than those given by the rate value 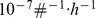 in our simulations, provides dCTP levels that are complementary to those of dFdC-TP. This indicates that the metabolic conditions, providing a sufficiently high propensity for the RR inhibition, give rise to dCTP and dFdC-TP levels that are complementary with respect to their relative amounts. In particular, for dCK inhibition regimes that result in dFdC-TP accumulation, the simulations show a corresponding complementary low level of dCTP accumulation due to optimal RR inhibition exerted by dFdC-DP. Similarly, for dCK inhibition regimes where dFdC-TP does not accumulate, we observe a high plateau for dCTP levels, resulting in low efficacy.

A decrease in dCK inhibition propensity has a significant positive effect to dFdC-TP accumulation for all the RR regimes. However at lower RR inhibition propensities, dCK inhibition propensity has a minor effect on the dCTP levels. As depicted in Figure 3, for the RR propensity regimes that do not result in a complementarity (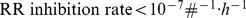), an increase in dCK inhibition propensity causes a depletion in the dFdC-TP pool, while dCTP demonstrates a minor tendency towards depletion which is subject to noise. An increase in dCK inhibition propensity causes a dFdC-TP accumulation, where dCTP demonstrates a tendency towards accumulation with noise. In contrast, the RR propensity regimes that result in a complementarity (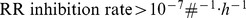) are more robust with respect to the noise in dCTP levels.

These observations indicate that in a continuum of non-efficacy to high-efficacy regimes, dCK inhibition propensity plays a role in determining dFdC-TP levels, whereas RR inhibition determines the complementarity of dFdC-TP and dCTP such that these metabolites complement each other with respect to their relative amounts.

### Sensitivity to RR and dCK inhibitions versus influx efficiency

It has been reported that efficient uptake is a requirement for gemcitabine efficacy, thus deficiency in nucleoside-transport is a possible mechanism of resistance [6], [10]. Indeed, experimental evidence indicates a significant correlation between gemcitabine chemotherapy outcome and human equilibrative nucleoside transporter-1 (hENT1) gene expression in pancreatic ductal adenocarcinoma [12]. In order to assess the effect of the efficiency in nucleoside transport in correlation with the inhibitory mechanism, we performed simulations where we varied the influx rate of dFdC, modeling variations in nucleoside-transporter expression levels in combination with the rates of the inhibitory mechanism.

**Figure 4:**
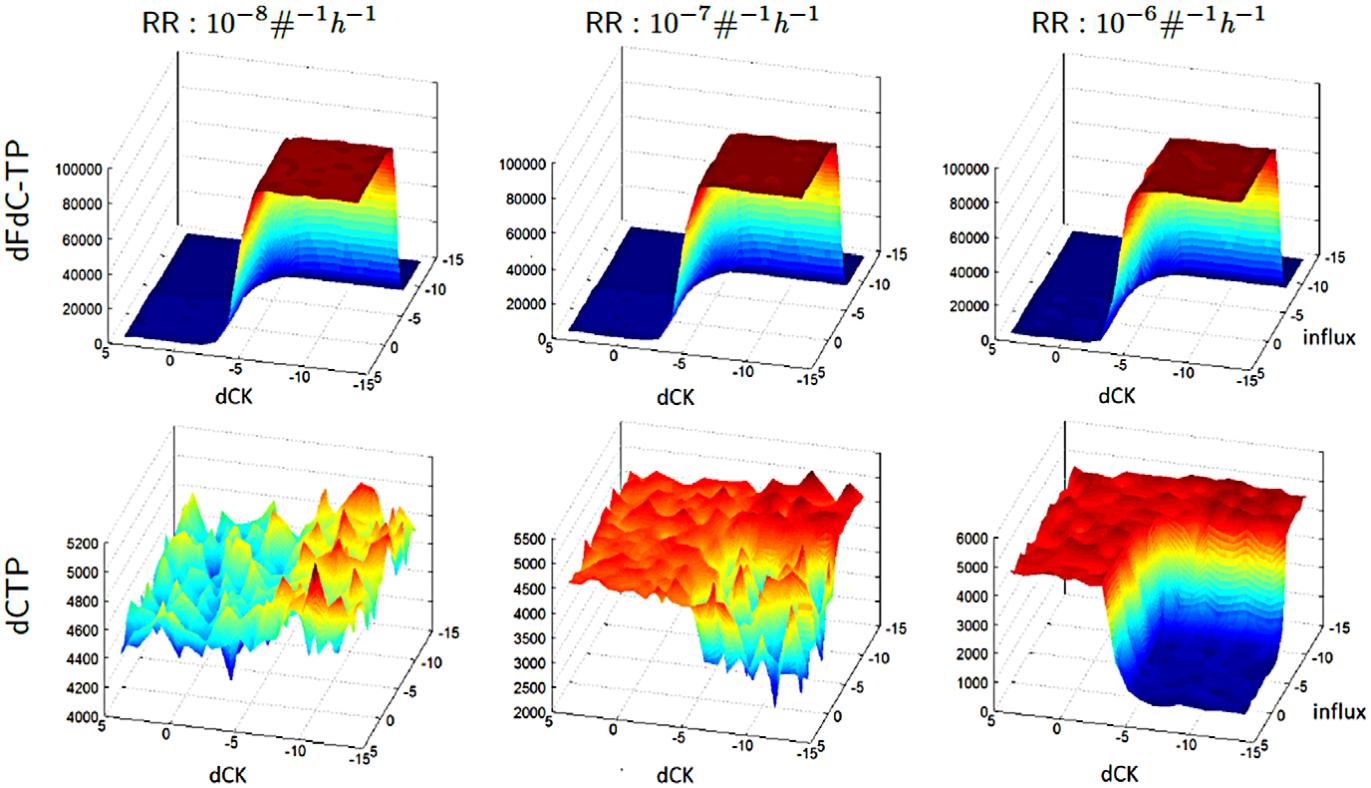
dCK and RR inhibitions versus influx. The plots displaying the area under the curve (AUC) for dFdC-TP (top row) and dCTP (bottom row) that result from the simulations with respect to different rate values for the RR and dCK inhibitions and the influx. At every column, there are plots for different RR inhibition association rates, varied from 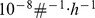 to 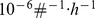 with an order of magnitude at each step. In each plot, the association rates of the dCK inhibition rate is varied from 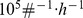 to 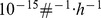, and the influx rate is varied from 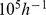 to 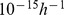, given in logarithmic scale. The influx rate 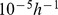 is the control regime used in the other simulations. The dissociation rates are set to 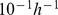. A scaling factor of 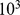 is used for intrinsic noise, which is observed at the dCTP plots, where RR inhibition rate values are less than 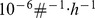.

In our simulations, at high dCK inhibition propensity regimes, dFdC-TP does not accumulate at all; this effect cannot be reverted by an increase in dFdC influx. The same results are observed with all tested RR inhibition regimes as depicted in Figure 4. When dCK inhibition is very low, influx propensity exerts a strong inducing effect on dFdC-TP accumulation that reaches its plateau very quickly. In all tested conditions of varying dCK inhibition and gemcitabine intake, the inhibition plays a major role in controlling both dFdC-TP and dCTP levels. However, dCTP accumulation is not greatly influenced by gemcitabine intake rate at low RR inhibition regimes. Increasing the RR inhibition propensity, the dCTP accumulation becomes highly sensitive to both dCK inhibition and gemcitabine influx. At RR inhibition of 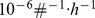 the dCTP and dFdC-TP complementary accumulation effect is fully restored.

Our simulations confirm that influx efficiency is essential for gemcitabine efficacy, implementing a switch-like mechanism: the rate value 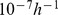 for influx is sufficient for the transformation of the dFdC into dFdC-TP, resulting in an immediate plateau, whereas an increase in influx efficiency does not provide a directly proportional increase in efficacy with respect to dFdC-TP accumulation. However, for certain regimes, corresponding to area around the rim of the dFdC-TP hills in Figure 4, deficiency in first phosphorylation of gemcitabine can be slightly compensated with a greater influx efficiency. Increased dFdC intake has no effect to dCTP accumulation for low RR inhibition propensities, while for greater propensities the complementarity between dCTP and dFdC-TP is preserved. Our model thus indicates that nucleoside transport efficiency is essential for efficacy, while with sufficient influx, gemcitabine efficacy with respect to dCTP depletion remains a function of RR inhibition. This also preserves the complementarity of the dCK and dFdC-TP metabolite levels, which require propensities for the RR inhibition that are greater than those given by the rate value 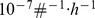 in order to maintain the robustness of the complementarity in our simulations.

## Discussion

Modeling and simulation of the interplay between metabolism and the pharmaceutical agents are gaining increasing attention in drug development due to their potential in reducing costs and accelerating the development process [39], [40]. In silico models promise the possibility of addressing preliminary queries regarding the interactions between the drug and the metabolic machinery by resorting to inexpensive resources. These models can provide better experimental design, and an improved understanding of clinical results. In this respect, gemcitabine is an ideal target for in silico experiments by models.

Based on experimental data by Veltkamp et al. [7], we have presented an algorithmic model of gemcitabine molecular machinery. Simulations with our model allowed us to quantify the gemcitabine efficacy, given by the AUC of dFdC-TP and dCTP metabolites in correlation with metabolic enzymes. AUC, which we use in our analysis, is a common measurement of efficacy. As in Veltkamp et al. 's analysis, it is used in our model as an index of the total exposure to the gemcitabine, and thereby provides an influence assessment of the inhibitory mechanisms within the metabolic network. In our model, CDP production can be extended to the previous steps of the cascade. We designed our model to capture the experimentally observed CDP availability, which is a requirement for the antagonistic behavior, given by the competition between dFdC-TP and dCTP. Other chemical agents that interfere with various parts of the machinery, e.g., tetrahydrouridine for inhibiting the effect of cytidine deaminase (CDA) and dCMPD, can be included as well.

Our model is a phenomenological model, as it is based on the knowledge of the processes in terms of structural connectivity and functional mechanisms. Alternatively pharmacological studies rely on empirical models, which are commonly used to describe and simulate single experiments in experimental or clinical pharmacokinetics [41]. In contrast to phenomenological models, these experiments consist in sampling biological fluids in order to measure the decline in drug concentration versus tumor size. Along these lines, Tham et al. [42] presented a kinetic-dynamic empirical model of gemcitabine-treated tumors in non-small-cell lung cancer patients. Using this model, the authors were able to predict the tumor size following treatment with gemcitabine. In this respect, a promising direction of investigation is the relationship between the phenomenological and the empirical models of gemcitabine, which can also help to better understand the relationships between the pharmacokinetics (that is, the mechanisms of absorption, distribution, and excretion) and the pharmacodynamics (that is, how the drug concentration is translated into a pharmacological effect) of gemcitabine.

In a related work, Battaglia et al. [43] developed a deterministic model of the intracellular metabolism of gemcitabine, coupled with a systemic pharmacokinetics model and a simplified cell cycle pharmacodynamics model. The model was fit to in vitro data collected by Heinemann et al [44] to estimate the parameters of gemcitabine triphosphate generation and elimination in leukemia cells. Stochasticity was introduced only through a randomization of the parameter vector to simulate inter-patient variability. Stochasticity of our model is helpful in capturing the intrinsic variability of the biochemical network [23]. In this respect, the choice of the stochastic framework is motivated by the intrinsic stochastic nature of the interactions governing the biochemical network responsible for the transformations of the drug. Recent studies indicate that stochastic models can reflect clinical data, as they take into account the intrinsic random fluctuations due to the variability of the environmental conditions caused by the differences in the genetic background of the patients [45]–[47]. In our model, by using scaling factors we alter the noise in the system, mimicking the variations in the environment under different regimes. While a scaling factor of 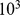 results in fluctuations in the AUC analysis in some cases, smaller scaling factors flatten out these fluctuations, hence the noise in the system.

Gemcitabine is broadly used in cancer therapy [5]. Because of the significant advances made by the experimental and theoretical studies about gemcitabine pharmacokinetics [48]–[52], detailed phenomenological models of the intracellular metabolism of gemcitabine can be built to enhance the understanding of its efficacy determinants. In this respect, an important question that is related to our model from a clinical point of view is the interplay between toxicity and efficacy, which requires a treatment of the system at the tissue level. The toxicity of gemcitabine is influenced by multiple factors. These include the interplay between the dosing schedule, and the first phosphorylation rate, which is a function of the amount of available dCK; the efficiency of cellular transport by hNTs; and the deamination to dFdU, which is influenced by the amount of available CDA. Moreover, the efficiency of the elimination kinetics of dFdC-TP plays a crucial role in modulating the levels of gemcitabine accumulation [2], [44]. A treatment of these factors together with genetic parameters specific to each person, for instance CDA genetic polymorphisms [2], [53], [54], within a tissue level consideration can provide the predictions on the interplay between toxicity and efficacy. From a clinical consideration, the main point of interest here is obtaining an estimate of the minimal amount of drug that is effective with minimal side effects. In this respect, the plots in Figures 2, 3, and 4 show that the AUC of dFdC-TP is a step-wise function of the enzyme concentrations and provide a quantification of the enzymes that are required to have a rapid significant increase of the AUC of dFdC-TP in conjunction with the subsequent saturation of the dCTP accumulation rate. As a consequence, given the dose of the drug, our model links the administered dose to the accumulation of the active metabolites. Specification and quantification of further aspects of the interplay between efficacy and the toxic effects can be achieved by considering cell populations within a loop of in silico modeling and clinical and wet lab experiments.

Our simulations predict the existence of a continuum of non-efficacy to high-efficacy regimes where the levels of the metabolites dFdC-TP and dCK are coupled in a complementary manner. While confirming that efficiency in transporter proteins is crucial for maintaining the high efficacy regimes, our model suggests that the complementarity of the dCK and dFdC-TP metabolite levels is a function of the association of dFdC-DP with RR. However, the levels of both dFdC-TP and dFdU-TP metabolites are affected by the inhibition due to the association of dCK and dCTP. Our model suggests that the three main resistance mechanisms are due to dCK deficiency, RR up-regulation upon gemcitabine administration, and decreased gemcitabine entry when nucleoside transporters' function is impaired. These three aspects should undergo clinical investigations with respect to their combined effects.

In this respect, clinical observations reported in [12] on expression levels of RR indicate that patients with ribonucleotide reductase regulatory subunit M1 (RRM1) above 1.00 gene expression ratio with GAPDH had a median time to progression, calculated from the date of diagnosis to the date of first progression or or last follow-up in metastatic patients, of 5.85 months compared with 13.30 and 9.92 months in patients with RRM1 below 1.00 and 0.95, respectively. Similar results are reported in [55] on nonsmall cell lung cancer patients with low expression of the RRM1, which significantly benefited from gemcitabine/cisplatin neoadjuvant chemotherapy. While resistance to gemcitabine was associated with both RRM1 and RRM2 overexpression [17], [56], small interfering RNA targeting the RRM2 catalytic subunit, reported in [57], enhanced the chemosensitivity to gemcitabine of pancreatic adenocarcinoma in vitro and in vivo.

As suggested by these results and our model, biochemical agents that contribute to concomitant low dCK inhibition and high RR inhibition propensities can be instrumental in overcoming resistance. Other experiments can involve agents that enhance RR inhibition to compensate for lower initial gemcitabine dose, and decrease the dCTP accumulation. This can in turn decrease dCK inhibition, favoring dFdC-TP accumulation. In patients with impaired entry, further inhibiting RR can also provide a mechanism to overcome resistance. With respect to these observations, our model, appropriately tailored on pharmacological and clinical measurements and observations, can be used as a tool to predict resistance or sensitivity in selected patient populations in specific circumstances.

## Materials and Methods

We use time-continuous discrete stochastic models, specified in the BlenX modeling language (BlenX [58], [59], COSBILAB MODEL – www.cosbi.eu). BlenX is explicitly developed to model biochemical entities and their interactions, and it is a part of the software platform COSBILAB that implements a modeling, analysis and simulation framework that is inspired by algorithmic systems biology [26]. BlenX is equipped with a stochastic simulation engine based on Gillespie algorithm [60]. In the model, the gemcitabine biochemical network is considered as a complex parallel information processing system [26]. The code of the model and its chemical reactions representation are provided in the supplementary material and in Figure S1.

While constructing the model, we made a number of assumptions: (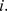) We simplified the distinction between the intracellular and extracellular deamination of gemcitabine by considering only intracellular deamination. This reflects a more general setting, since extracellular deamination strongly depends on the nature of the tissue in consideration. (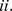) For the enzymes, which do not participate in an inhibition, we simplified by assuming their constant concentration and factoring their amounts into the reaction rates. (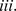) The activity in nucleoside-transport, e.g., due to human equilibrative/concentrative nucleoside transporters, is represented by influx and efflux rates by relying on mass action kinetics. This way, variations in rates provide an implicit representation of the variations in the expression levels of the transport proteins, which are manifested during simulation as propensities. (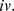) The indirect competition between the cascades that result in dFdC-TP and dCTP is implicitly encoded by means of the mass action dynamics that chooses an action at every simulation step due to underlying continuous time Markov chain semantics. The direct competition mechanisms between these cascades are given by means of the inhibitions between metabolites. In the experiments, where we vary the rates of the inhibitory mechanisms, we rely on mass action kinetics to implicitly implement various metabolic conditions that effect the levels of the participating metabolites.

**Figure 5:**
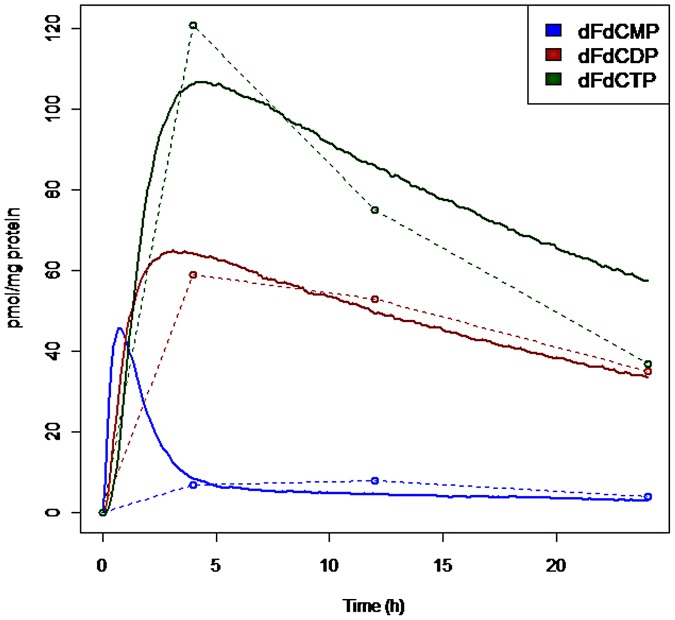
A sample simulation with the gemcitabine metabolization pathway. A sample gemcitabine metabolization pathway simulation is compared with the experimental data. The dashed lines connect the data points given by the experiments in [7].

For the calibration of the model, we used the time series data of the gemcitabine metabolite concentration measurements at experiments provided by Veltkamp et al. [7]. In these experiments, the concentrations of the intracellular metabolites have been measured in human hepatocellular carcinoma (HepG2) at four time points (0, 4, 12, and 24 hours). The measurements are reported in Table 1 in units of pico-moles/mg of cellular protein [7]. Because in HepG2 cells, 1 mg of cellular protein corresponds to 12
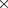
10^6^ cells with a volume of 17fL per cell [7], in the model there is an average of 8.33
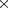
10^−8^ mg of protein per cell. For the estimation of the parameters of the gemcitabine metabolization cascade without the inhibitors, given in Table 2, we used the Nelder-Mead least squares method [61] implemented in the Systems Biology Toolbox 2 for Matlab [62], and obtained an agreement between experimental time series and simulations. Figure 5 provides a representative simulation output in comparison with the experimental data, where different simulations differ only in minor fluctuations due to the stochastic simulations.

**Table 1:** Intracellular concentration of observed metabolites.

Time (h)	dFdC	dFdCMP	dFdCDP	dFdCTP	dFdU	dFdUMP	dFdUDP	dFdUTP
0	0	0	0	0	0	0	0	0
4	3	7	59	121	0.08	1.12	0.68	1.34
12	1	8	53	75	0.06	1.15	0.41	0.66
24	0.1	4	35	37	0.03	0.2	0.07	0.2

The values have been taken from the concentration profiles reported in [?], where the units are given in pico-moles/mg of cellular protein.

**Table 2:** Estimated parameters.

reaction	rate	unit		reaction	rate	unit
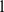	9.97234	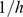		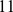	0.00000968	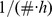
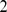	0.000261675	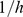		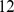	5.60415E-10	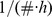
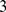	4.72336E-06	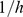		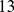	7.844E-07	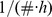
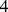	0.0508194	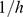		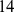	4.20541E-08	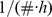
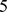	1.04994E-05	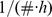		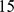	1.64322E-06	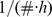
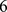	8.75208E-07	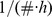		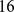	9.05139E-10	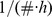
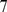	2.37162E-05	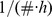		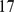	4.76746E-09	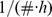
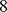	2.12216E-06	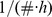		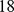	4.559E-08	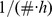
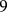	2.52037E-05	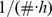		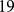	0.0544456	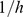
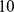	1.44908E-05	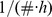		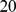	0.000737496	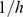

Because we used stochastic simulations, the time series of metabolite concentrations are converted into time series of numbers of molecules. With respect to the experiments, the initial extracellular concentration of dFdC (i.e., dFdCout) per cell is unknown. We estimated this value by assuming that within the first 4 hours half-life is negligible, and the metabolites of concern remain inside the cell. This way, the initial amount of gemcitabine outside can be approximated as the sum of all the metabolites at the 4th hour, that is, in the order of 10 million molecules. According to these considerations, the quantity of metabolites in number of molecules is given in Table 3.

**Table 3:** Observed number of molecules of intracellular metabolites.

Time (h)	dFdCout	dFdC	dFdCMP	dFdCDP	dFdCTP
0	9.70E+06	0	0	0	0
4	0	1.51E+05	3.51E+05	2.96E+06	6.07E+06
12	0	5.02E+04	4.01E+05	2.66E+06	3.76E+06
24	0	5.02E+03	2.01E+05	1.76E+06	1.86E+06

Time (h)	dFdUout	dFdU	dFdUMP	dFdUDP	dFdUTP
0	0	0	0	0	0
4	0	4.01E+03	5.62E+04	3.41E+04	6.72E+04
12	0	3.01E+03	5.77E+04	2.06E+04	3.31E+04
24	0	1.51E+03	1.00E+04	3.51E+03	1.00E+04

Number of molecule obtained by a conversion of the concentration values given in Table 1.

For the parameters of the cascade that results in the integration of dCTP to DNA, following [63], we took the average volume of cells as 0.943 pl/cell and cellular concentration of dCTP as 38.4 microM, and estimated the production rate of CDP such that the average number of molecules of dCTP is about 215,000 as reported in [7].

**Figure 6:**
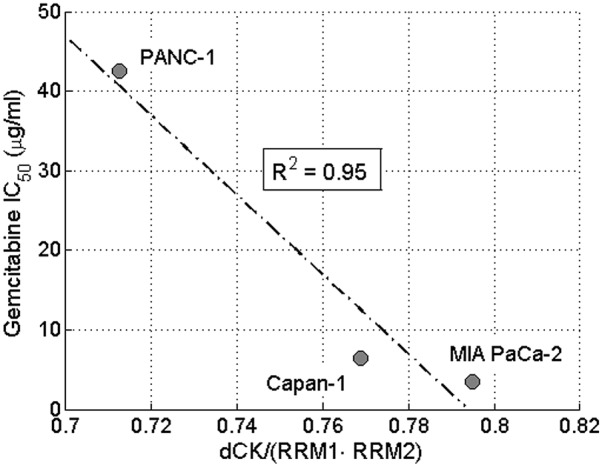
The correlation between gemcitabine efficacy and the ratio of dCK and RR. The correlation between gemcitabine efficacy, measured as 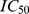 and the ratio of dCK and RR concentrations, given with 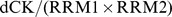, with respect to the experiments reported in [27].

**Figure 7:**
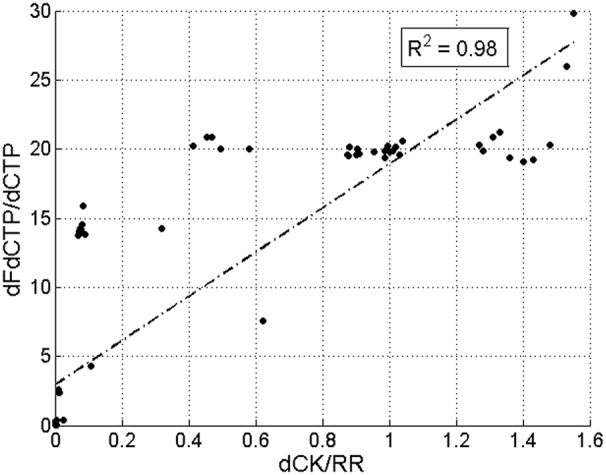
The correlation between gemcitabine efficacy and the ratio of dCK and RR in the simulations. The correlation between gemcitabine efficacy measured as 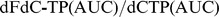 and the ratio of dCK and RR, given with 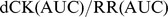 with respect to simulations with our model.

To validate the sensitivity analysis with respect to the parameters of the inhibitory mechanism, we have produced a set of different experimental conditions, which provide varying dCK and RR levels during simulations. By relying on the AUC ratio of 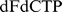 and 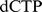 as the efficacy metric of our model, we compared our simulations with the experimental data in [27], depicted in Figure 6. In agreement with the experimental data, our model indicates that efficacy is proportional with the ratio of dCK and RR levels as depicted in Figure 7.

## Supporting Information

Figure S1
**Model reactions.** Reactions 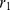-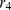 model the transport through the membrane, resulting in influx and efflux. Reactions 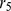-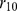 model the transformation of dFdC to its metabolites. Reactions 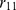-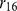 model the transformation of dFdU to its metabolites. Reactions 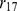 and 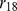 model the deamination of gemcitabine. Reactions 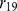 and 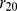 model the incorporation of dFdC-TP and dFdU-TP into DNA. Reactions 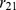-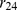 model the cascade that results in the incorporation of dCTP into DNA. Reactions 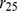-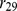 model the inhibitory mechanism.(TIF)

Figure S2
**The plots of the dFdC-TP AUCs resulting from the simulations where the association and dissociation rates of the inhibitions are varied from** 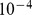 **to** 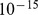 **, given in logarithmic scale.** The plots are from left to right for the dCK, dCMPD, and RR inhibitions. For the RR inhibition, only association rates are considered.(TIF)

File S1 **A description of the BlenX modeling language and the source code of the model.**
(PDF)
