# Supplementary figures and images for: Algorithmic Modeling Quantifies the Complementary Contribution of Metabolic Inhibitions to Gemcitabine Efficacy
Source: PLoS One. 2012 Dec 11;7(12):e50176. doi: 10.1371/journal.pone.0050176 (PMC3519828; doi:10.1371/journal.pone.0050176)

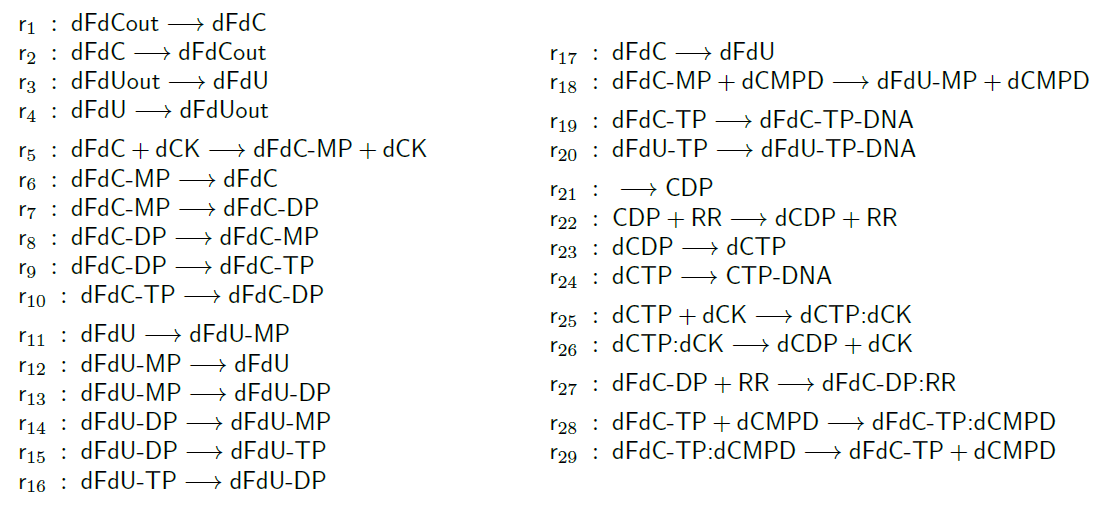

Supplement: Figure S1 — Model reactions. Reactions - model the transport through the membrane, resulting in influx and efflux. Reactions - model the transformation of dFdC to its metabolites. Reactions - model the transformation of dFdU to its metabolites. Reactions and model the deamination of gemcitabine. Reactions and model the incorporation of dFdC-TP and dFdU-TP into DNA. Reactions - model the cascade that results in the incorporation of dCTP into DNA. Reactions - model the inhibitory mechanism. (TIF) [file pone.0050176.s001.tif]

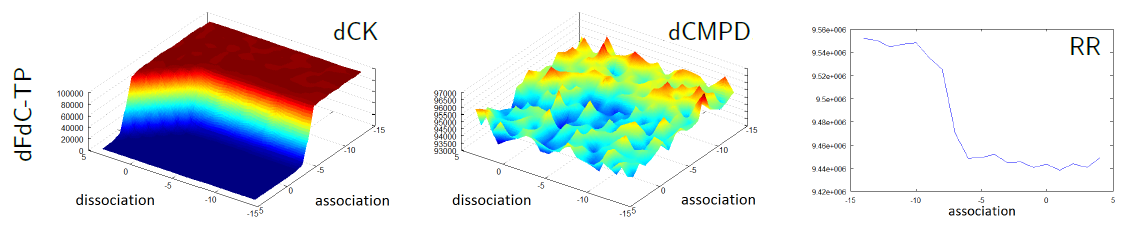

Supplement: Figure S2 — The plots of the dFdC-TP AUCs resulting from the simulations where the association and dissociation rates of the inhibitions are varied from to , given in logarithmic scale. The plots are from left to right for the dCK, dCMPD, and RR inhibitions. For the RR inhibition, only association rates are considered. (TIF) [file pone.0050176.s002.tif]
