# Supplementary material for: Algorithmic Modeling Quantifies the Complementary Contribution of Metabolic Inhibitions to Gemcitabine Efficacy
Source: PLoS One. 2012 Dec 11;7(12):e50176. doi: 10.1371/journal.pone.0050176 (PMC3519828; doi:10.1371/journal.pone.0050176)
Supplement: File S1 — A description of the BlenX modeling language and the source code of the model. (PDF) [file pone.0050176.s003.pdf]

1 BlenX Modeling Language

BlenX is a stochastic programming language, equipped with algebraic constructs that are instrumental for expressing concurrent interacting processes. Due to algebraic composability properties, a BlenX model can be composed from simple modules, and can be easily extended when new data and new biological knowledge are acquired.

In BlenX, each species is given with an abstract entity called a *box*. Each box has a number of connectivity interfaces called *binders*, and it is equipped with an internal program. A box can interact with another box, and change state as a result of this interaction with respect to the actions specified in its internal program. Alternatively, a box can autonomously change state by stochastically performing an action that is given in its internal program. The sites of interaction are represented as binders on the box surface.

The internal program of a box determines the *actions* a box can undertake. These actions are summarized as follows: a box can (*i.*) communicate with another box that is bound to it (or with itself) by performing an input action, e.g., `x?(message)` that is complementary to the output action, e.g., `x!(message)`, of the other box, or vice versa, and this way send or receive a message; (*ii.*) perform a stochastic `delay` action; (*iii.*) change (`ch`) the type of one of its interfaces; (*iv.*) eliminate itself by performing a `die` action; (*v.*) `expose` a new binder; (*vi.*) `hide` one of its binders; (*vii.*) `unhide` a binder which is hidden; (*viii.*) do nothing by executing the `nil` action. In addition to these actions, there are also other programming constructs available such as if-then statements and state-checks.

In BlenX, following the process algebra tradition, we can compose actions by using algebraic composition operators to define increasingly complex behaviors. We can sequentially compose actions by resorting to the prefix-operator, which is written as an infix dot. For instance, `y?().ch(y,C).nil` denotes a program that first performs an input action and then a change action. Programs can be composed in parallel. Parallel composition (denoted by the infix operator `|`, for instance `P|Q`) allows the description of programs, which may run independently in parallel and also synchronize on *complementary actions* (i.e., *input* and *output* over the same channel). Programs can also be composed by *choice*, denoted with the summation operator "+". The sum of processes `P` and `Q`, `P + Q` behaves either as `P` or as `Q`, determined by their rates, and selection of one discards the other forever. Another programming construct that is available in BlenX is *events*. Events are used for expressing actions that are enabled by global conditions, for example, to model the production of a species at a certain time during the simulation. Among other features of process-algebra-based languages that provide composability and the ability to model complex structures, these features constitute a kind of modularity in the construction of the models.

BlenX Code - prog file.

```
[ time = 24 ] // time in h

<< BASERATE : inf >>

// MAIN PROCESS

let p_main : pproc =
if (s, c_dFdCout) then
ch(rate(r_dFdCout_trans_in), s, c_dFdC).start_p_main!() +
ch(rate(r_dFdCout_CDA), s, c_dFdUout).start_p_main!()
endif +
if (s, c_dFdUout) then
ch(rate(r_dFdUout_trans_in), s, c_dFdU).start_p_main!()
```

```
endif +
if (s, c_dFdC) then
ch(rate(r_dFdC_trans_out), s, c_dFdCout).start_p_main!() +
s?().ch(s, c_dFdCMP).start_p_main!() +
ch(rate(r_dFdC_CDA), s, c_dFdU).start_p_main!()
endif +
if (s, c_dFdCMP) then
ch(rate(r_dFdCMP_NMPK), s, c_dFdCDP).start_p_main!() +
ch(rate(r_dFdCMP_P1), s, c_dFdC).start_p_main!()+
s?().ch(s, c_dFdUMP).start_p_main!()
endif +
if (s, c_dFdCDP) and not(s, bound) then
ch(rate(r_dFdCDP_NDPK), s, c_dFdCTP).start_p_main!() +
ch(rate(r_dFdCDP_P2), s, c_dFdCMP).start_p_main!()
endif +
if (s, c_dFdCTP) and not(s, bound) then
ch(rate(r_dFdCTP_P3), s, c_dFdCDP).start_p_main!() +
ch(rate(r_dFdCTP_DNA), s, c_dFdCTPDNA)
endif +
if (s, c_dFdU) then
ch(rate(r_dFdU_trans_out), s, c_dFdUout).start_p_main!() +
ch(rate(r_dFdU_TK1), s, c_dFdUMP).start_p_main!()
endif +
if (s, c_dFdUMP) then
ch(rate(r_dFdUMP_NMPK), s, c_dFdUDP).start_p_main!() +
ch(rate(r_dFdUMP_P1), s, c_dFdU).start_p_main!()
endif +
if (s, c_dFdUDP) then
ch(rate(r_dFdUDP_NDPK), s, c_dFdUTP).start_p_main!() +
ch(rate(r_dFdUDP_P2), s, c_dFdUMP).start_p_main!()
endif +
if (s, c_dFdUTP) then
ch(rate(r_dFdUTP_P3), s, c_dFdUDP).start_p_main!() +
ch(rate(r_dFdUTP_DNA), s, c_dFdUTPDNA)
endif ;


// GEMCITABINE AND ITS METABOLITES
// (dFdC, dFdCMP, dFdCDP, AND dFdCTP)

let dFdCout: bproc = #(s, c_dFdCout)
  [ p_main | rep start_p_main?().p_main ];

let dFdC   : bproc = #(s, c_dFdC)
  [ p_main | rep start_p_main?().p_main ];

let dFdCMP : bproc = #(s, c_dFdCMP)
  [ p_main | rep start_p_main?().p_main ];
```

```
let dFdCDP : bproc = #(s, c_dFdCDP)
  [ p_main | rep start_p_main?().p_main ];

let dFdCTP : bproc = #(s, c_dFdCTP)
  [ p_main | rep start_p_main?().p_main ];

let dFdCTPDNA : bproc = #(s, c_dFdCTPDNA)
  [ rep start_p_main?().p_main ];

// GEMCITABINE DEAMINATED AND ITS METABOLITES
// (dFdU, dFdUMP, dFdUDP, AND dFdUTP)

let dFdUout: bproc = #(s, c_dFdUout)
  [ p_main | rep start_p_main?().p_main ];

let dFdU   : bproc = #(s, c_dFdU)
  [ p_main | rep start_p_main?().p_main ];

let dFdUMP : bproc = #(s, c_dFdUMP)
  [ p_main | rep start_p_main?().p_main ];

let dFdUDP : bproc = #(s, c_dFdUDP)
  [ p_main | rep start_p_main?().p_main ];

let dFdUTP : bproc = #(s, c_dFdUTP)
  [ p_main | rep start_p_main?().p_main ];

let dFdUTPDNA : bproc = #(s, c_dFdUTPDNA)
  [ rep start_p_main?().p_main ];


// CYTIDINE DIPHOSPHATE (CDP, dCDP, AND dCTP)

let p_CDP : pproc =
if (s, c_CDP) then
s?().ch(s, c_dCDP).start_p_CDP!()
endif +
if (s, c_dCDP) then
ch(rate(r_dCDP_NDPK), s, c_dCTP).start_p_CDP!() +
ch(rate(r_dCDP_P4), s, c_CDP).start_p_CDP!()
endif +
if (s, c_dCTP) and not(s, bound) then
ch(rate(r_dCTP_P5), s, c_dCDP).start_p_CDP!() +
ch(rate(r_dCTP_DNA), s, c_dCTPDNA)
endif ;

let CDP : bproc = #(s, c_CDP)
  [ p_CDP | rep start_p_CDP?().p_CDP ];
```

```
let dCDP : bproc = #(s, c_dCDP)
  [ p_CDP | rep start_p_CDP?().p_CDP ];

let dCTP : bproc = #(s, c_dCTP)
  [ p_CDP | rep start_p_CDP?().p_CDP ];

let dCTPDNA : bproc = #(s, c_dCTPDNA)
  [ rep start_p_CDP?().p_CDP ];


// DEOXYCYTIDINE KINASE (dCK)

let dCK : bproc = #(s, c_dCK)
  [ s!().startdCK!() | rep startdCK?().s!().startdCK!() ];


// RIBONUCLEOTIDE REDUCTASE (RR)

let RR : bproc = #(s, c_RR)
  [ s!().startRR!() | rep startRR?().s!().startRR!() ];


// ALTRO ENZIMA (dCMPD)

let dCMPD : bproc = #(s, c_dCMPD)
  [ s!().startdCMPD!() | rep startdCMPD?().s!().startdCMPD!() ];


// INHIBITION COMPLEXES

let dCTP_dCK_inhibition : complex  = {
((Box1, s, Box2, s));
Box1 : dCTP = (s);
Box2 : dCK = (s);
};

let dFdCDP_RR_inhibition : complex  = {
((Box1, s, Box2, s));
Box1 : dFdCDP = (s);
Box2 : RR = (s);
};

let dFdCTP_dCMPD_inhibition : complex  = {
((Box1, s, Box2, s));
Box1 : dFdCTP = (s);
Box2 : dCMPD = (s);
};
```

```
// CDP PRODUCTON

when (CDP : : rate(r_CDP_inj)) new(1);

run dFdCMolecules dFdCout || dCKMolecules dCK ||
    EnzymeMolecules dCMPD || CDPMolecules CDP || RRMolecules RR
```

BlenX Code - types file.

```
{
    // GEMCITABINE AND ITS PHOSPHATES (dFdC, dFdCMP, dFdCDP, AND dFdCTP)
    c_dFdCout, c_dFdC, c_dFdCMP, c_dFdCDP, c_dFdCTP, c_dFdCTPDNA,

    // GEMCITABINE deamination (and phosphates dFdU, dFdUMP, dFdUDP, AND dFdUTP)
    c_dFdUout, c_dFdU, c_dFdUMP, c_dFdUDP, c_dFdUTP, c_dFdUTPDNA,

    // DEOXYCYTIDINE KINASE (dCK)
    c_dCK,

    // RIBONUCLEOTIDE REDUCTASE (RR)
    c_RR,

    //dCMPD
    c_dCMPD,

    // CYTIDINE DIPHOSPHATE (CDP, dCDP, AND dCTP)
    c_CDP, c_dCDP, c_dCTP, c_dCTPDNA
}
%%
{
    (c_dFdC, c_dCK, rate(r_dFdC_dCK)),
    // dFdC + dCK -> dFdCMP + dCK

    (c_dFdCMP, c_dCMPD, rate(r_dFdCMP_dCMPD)),
    // dFdCMP + dCMPD -> dFdUMP + dCMPD

    (c_CDP, c_RR, rate(r_CDP_RR)),
    // CDP + RR -> dCDP + RR

    // INHIBITIONS
    (c_dFdCDP, c_RR, rate(r_dFdCDP_RR_bin), rate(r_dFdCDP_RR_unb), 0),
    // dFdCDP inhibits RR

    (c_dFdCTP, c_dCMPD, rate(r_dFdCTP_dCMPD_bin), rate(r_dFdCTP_dCMPD_unb), 0),
    // dFdCTP inhibits dCMPD

    (c_dCTP, c_dCK, rate(r_dCTP_dCK_bin), rate(r_dCTP_dCK_unb), 0)
    // dCTP inhibits dCK
}
```

BlenX Code - func file.

```
// SCALING FACTOR
let sf : const = 1000;

// INITIAL CONDITIONS (Number of molecules)
let dFdCMolecules : const = 10000000/sf;
let CDPMolecules : const = 200000/sf;
let EnzymeMolecules : const = 100000/sf;
let dCKMolecules : const = 100000/sf;
let RRMolecules : const = 100000/sf;

let EMsf : const = EnzymeMolecules*sf;

// RATES

// ----------- OUTSIDE THE CELL -----------

// TRANSPORT
let r_dFdCout_trans_in : const =  0.0000997234*EMsf;
let r_dFdC_trans_out : const = 0.00000000261675*EMsf;
let r_dFdUout_trans_in : const = 0.0000000000472336*EMsf;
let r_dFdU_trans_out : const = 0.000000508194*EMsf;

// DEAMINATION OF dFdC
let r_dFdCout_CDA : const = 0*EMsf;

// ------------ INSIDE THE CELL ------------

// PHOSPHORYLATION AND DEPHOSPHORYLATION OF dFdC AND dFdU
let r_dFdC_dCK : const = 0.0000104994*sf;
let r_dFdCMP_P1 : const = 0.000000875208*EMsf;
let r_dFdCMP_NMPK : const = 0.0000237162*EMsf;
let r_dFdCDP_P2 : const = 0.00000212216*EMsf;
let r_dFdCDP_NDPK : const = 0.0000252037*EMsf;
let r_dFdCTP_P3 : const = 0.0000144908*EMsf;
let r_dFdU_TK1 : const = 0.0000000000968*EMsf;
let r_dFdUMP_P1        : const = 0.000000000560415*EMsf;
let r_dFdUMP_NMPK : const = 0.0000007844*EMsf;
let r_dFdUDP_P2 : const = 0.0000000420541*EMsf;
let r_dFdUDP_NDPK : const = 0.00000164322*EMsf;
let r_dFdUTP_P3 : const = 0.000000000905139*EMsf;

// DEAMINATION OF dFdC
let r_dFdC_CDA : const = 0.00000000476746*EMsf;
let r_dFdCMP_dCMPD : const = 0.00000004559*sf;

// INJECTION, DEOXIDATION, AND OXIDATION OF CDP
let r_CDP_inj : const = 1e5/sf;
let r_CDP_RR : const = 0.0005*sf;
```

```
let r_dCDP_P4 : const = 0*EMsf;

// PHOSPHORYLTAION AND DEPHOSPHORYLATION OF dCDP
let r_dCDP_NDPK : const = 0.000252037*EMsf;
let r_dCTP_P5 : const = 0*EMsf;

// INHIBITIONS
let r_dFdCDP_RR_bin : const = 1e-7*sf;
let r_dFdCDP_RR_unb : const = 1e-1;
let r_dCTP_dCK_bin : const = 1e-7*sf;
let r_dCTP_dCK_unb : const = 1e-1;
let r_dFdCTP_dCMPD_bin : const = 1e-7;
let r_dFdCTP_dCMPD_unb : const = 1e-1;

// DNA INCORPORATION
let r_dFdCTP_DNA : const = 0.000000544456*EMsf;
let r_dFdUTP_DNA : const = 0.00000000737496*EMsf;
let r_dCTP_DNA : const = 0.000005*EMsf;
```
